# Photodynamic Therapy with Hypericin Improved by Targeting HSP90 Associated Proteins

**DOI:** 10.3390/ph4111488

**Published:** 2011-11-10

**Authors:** Peter Solár, Mária Chytilová, Zuzana Solárová, Ján Mojžiš, Peter Ferenc, Peter Fedoročko

**Affiliations:** 1 Laboratory of Cell Biology, Institute of Biology and Ecology, Faculty of Sciences, P.J. Šafárik University, 040 01 Košice, Slovak Republic; E-Mails: peter.solar@upjs.sk (P.S.); mariachytilova@yahoo.com (M.C.); peter.ferenc@upjs.sk (P.F.); 2 Geriatric Nursing Clinic, Faculty of Medicine, P.J. Šafárik University, 040 01 Košice, Slovak Republic; E-Mail: zuzana.solarova@upjs.sk; 3 Department of Pharmacology, Faculty of Medicine, P.J. Šafárik University,040 01 Košice, Slovak Republic; E-Mail: jan.mojzis@upjs.sk

**Keywords:** hypericin-PDT, 17-DMAG, HSP90

## Abstract

In this study we have focused on the response of SKBR-3 cells to both single 17-DMAG treatment as well as its combination with photodynamic therapy with hypericin. Low concentrations of 17-DMAG without any effect on survival of SKBR-3 cells significantly reduced metabolic activity, viability and cell number when combined with photodynamic therapy with hypericin. Moreover, IC_10_ concentation of 17-DMAG resulted in significant increase of SKBR-3 cells in G1 phase of the cell cycle, followed by an increase of cells in G2 phase when combined with photodynamic therapy. Furthermore, 17-DMAG already decreased HER2, Akt, P-Erk1/2 and survivin protein levels in SKBR-3 cells a short time after its application. In this regard, 17-DMAG protected also SKBR-3 cells against both P-Erk1/2 as well as survivin upregulations induced by photodynamic therapy with hypericin. Interestingly, IC_10_ concentration of 17-DMAG led to total depletion of Akt, P-Erk1/2 proteins and to decrease of survivin level at 48 h. On the other hand, 17-DMAG did not change HER2 relative expression in SKBR-3 cells, but caused a significant decrease of HER2 mRNA in MCF-7 cells characterized by low HER2 expression. These results show that targeting HSP90 client proteins increases the efficiency of antineoplastic effect of photodynamic therapy *in vitro*.

## 1. Introduction

The HER2 oncoprotein is an important therapeutic target in the treatment of invasive breast cancers associated with poor disease-free survival and resistance to chemotherapy [1]. HER2 status is a significant prognostic factor for local-regional disease progression. Patients with positive HER2 status had a local-regional disease progression-free rate of 59%, compared with 92% for patients with negative HER2 status [2].

Although the application of monoclonal antibody against HER2-trastuzumab has shown beneficial effects when combined with docetaxel and platinum salts [3] or paclitaxel and carboplatin [4,5], its use beyond first-line therapy might develop resistance to this agent. In this regard, inhibition of PTEN [6], overexpression of IGF-IR [7] and MUC4 [8] and increased level of VEGF protein [9] could play a significant role. In order to make trastuzumab treatment more effective after disease progression, new agents targeting the HER2 pathway have been developed. HER-targeting agents include antibodies, small tyrosine kinase inhibitor (TKI) molecules, inhibitors of mTOR, farnesyltransferase, PI3K and HSP90 inhibitors [10].

Nowadays, the natural inhibitors radicicol, geldanamycin and its derivatives (17-AAG, 17-DMAG, IPI-504, IP-493) as well as several synthetic inhibitors are reported to block nucleotide binding of HSP90 molecule and result in ubiquitination and proteasomal degradation of HSP90 client proteins. HER2 receptor is one of the most sensitive client proteins to geldanamycin and its derivatives which was shown by many experimental and clinical studies [11-17].

An alternative method, at least for chest wall recurrence of breast carcinoma, is photodynamic therapy (PDT). The ground-state photosensitizers such as porphyrins, chlorophylls and some dyes administered locally or systemically in PDT accumulate preferentially in tumor tissue [18] and after subsequent exposure to a light source of suitable wavelength are excited and can subsequently react through a Type I free radical mechanism or alternatively via a Type II reaction involving reactive singlet oxygen [19]. Allison *et al.* [20] have used PDT to control recurrent breast cancer that has failed to respond to conventional therapy. PDT offers patients with chest wall progression a treatment option with an excellent clinical response and allows opportunities for good long-term local tumor control [21].

Our recent *in vitro* study showed a decline in HER2 mRNA levels a short time after photoactivation of hypericin in SKBR-3 cells, but no changes in HER2 mRNA were found in dark conditions [22]. Furthermore, we have also demonstrated hypericin-PDT mediated degradation of HER2 receptor in the same cell line via lysosomal activity [23]. These results as well as known facts about HSP90-directed agent benzoquinone ansamycin-geldanamycin led us to determine the effect of combination of PDT and HSP90 inhibitor on the response of HER2 overexpressed SKBR-3 cells *in vitro*. We hypothesized that degradation of HSP90 client proteins induced by HSP90 inhibitor could be beneficial for hypericin-PDT mediated anti-tumor activity. Instead of geldanamycin we used a less toxic and more soluble form of geldanamycin – 17-dimethylaminoethylamino-17-demethoxygeldanamycin (17-DMAG).

## 2. Experimental Section

### 2.1. Cell Culture and Treatments

Human breast adenocarcinoma cell lines SKBR-3 and MCF-7 (kindly provided by the Institute of Biophysics, Czech Academy of Sciences, Brno, Czech Republic) were cultured in McCoy and RPMI-1640 medium, respectively and maintained at 37 °C in a humidified 5% CO_2_ atmosphere, protected from light. Media were supplemented with 10% fetal calf serum (FCS), antibiotics (penicillin 100 U/mL, streptomycin 100 μg/mL and amphotericin 25 μg/mL; Invitrogen Co., Carlsbad, CA, USA).

The cells were incubated with different concentrations (0.84–210 nM) of hypericin (AppliChem, Darmstadt, Germany) and/or 17-DMAG (0.5–500 nM) (Alexis Biochemical, Lausen, Switzerland) in dark conditions for 24 h followed by photosensitization (for details see below) and further 24 or 48 h incubation of cells in a humidified 5% CO_2_ atmosphere, protected from light. For hypericin-PDT + 17-DMAG treatment 5 nM concentration of 17-DMAG inhibiting 10% of cells metabolic activity (IC_10_; determined based on MTT assay) was chosen. In this regard, IC_50_ concentration of 17-DMAG was 19 nM.

### 2.2. Cell Photosensitization

The cells were irradiated by placing the experimental flasks on a plastic diffuser sheet above a set of eleven L18W/30 lamps (Osram, Berlin, Germany) with the maximum emission between 530 and 620 nm (the absorption peak of hypericin is 595 nm). Uniform fluency rate of about 4.4 mW/cm^2^s (measured by TES 1335 luxmeter (Rotronic, Taipei, Taiwan) and the temperature not exceeding 37 °C at the surface of the diffuser were measured. The light dose (4.4 J/cm^2^) was calculated by multiplying the fluency rate by exposure time (16 min 40 s).

### 2.3. Cell Number and Viability

The number of cells was determined using Coulter Counter (Model ZF, Coulter Electronics Ltd., Luton, BEDS., UK) and the total viability was analyzed by staining of cells with 0.15% eosin via light microscopy.

### 2.4. Cell Cycle Analysis

The distribution of cells at different stages in the cell cycle was estimated by flow cytometric DNA analysis. Cells were harvested, washed with phosphate-buffered saline (PBS), fixed in 70% ice cold ethanol and stored at 4 °C for 24 h. Fixed cells were centrifuged, washed with PBS, stained with staining solution (20 μg/mL propidium iodide, 137 μg/mL RNase A and 0.1% Triton X-100 in PBS) in the dark for 30 min and measured with a flow-cytometer (FACSCalibur, Becton Dickinson, San Diego, CA, USA). For each sample, a minimum of 15,000 cells was evaluated and analyzed using ModFit LT 3.0 software (Verity Software House, Topsham, ME, USA). Cells characterized by DNA content lower than diploid (subG0/G1 population) were considered as apoptotic cells.

### 2.5. Clonogenic Assay

SKBR-3 cells (2.5 × 10^5^ cells/mL) settled in Petri dishes (60 mm diameter) were incubated for 24 h after hypericin photoactivation, subsequently harvested by trypsinization and washed twice with fresh medium. Viable cells numbering 1,000/well were then seeded into a 6-well tissue culture plate and allowed to grow for 10 days in culture conditions until visible colonies (more than 50 cells per colony) were observed. The cells were then stained with 0.1% methylene blue dye in 80% ethyl alcohol, and the visible colonies (more than 50 cells/colony) were counted using Clono-Counter software [24]. The results of the clonogenic assay are presented as means ± SD of three independent experiments

### 2.6. MTT Assay

The cells were seeded into 96-well cell culture plates at a density of 5 × 10^3^ SKBR-3 cells per well. MTT (3-(4.5-dimethylthiazol-2-yl)-2.5-diphenyl tetrazolium bromide; Sigma Chemicals Co., St. Louis, MO, USA) was added in the final concentration of 0.2 mg/mL at 24 h after hypericin photoactivation, followed by 4 h cell incubation at 37 °C and solubilization of MTT-formazan product using 3.3% sodium dodecyl sulfate (SDS, Sigma Chemicals Co.). The absorbance measurements were carried out using a FLUOstar Optima universal microplate reader (BMG Labtechnologies GmbH, Offenburg, Germany) and expressed as a percentage of the dye extracted from untreated control cells ([OD value of treated cells/mean OD value of control cells] × 100%).

### 2.7. Western Blotting

Western blot analyses were carried out according to the standard protocol. The protein sample was separated on 10% SDS-PAGE, electroblotted onto Immobilon-P transfer membrane (Millipore Co., Billerica, MA, USA) and incubated using primary antibodies shown below: anti-HER2 (sc-284, 1:200), anti-survivin (sc-8807, 1:200; Santa Cruz Biotechnology, CA, USA), anti-HSP90 (ALX-804-808, 1:1,000; Alexis Biochemical), anti-Akt (#9272, 1:1,000), anti-p44/42 MAP kinase (#9102, 1:1,000), anti-phospho-p44/42 MAP kinase (#9272, 1:1,000) and anti-β-actin (clone AC-74, 1:10,000; Sigma Chemicals Co.). Subsequently, the membranes were incubated with secondary horseradish peroxidase-conjugated antibodies (Goat anti-Rabbit IgG F(AB') 2, 1:10,000, PI-31461, Goat anti-Mouse IgG F(AB') 2, 1:10,000, PI-31436 or Rabbit anti-Goat IgG F(AB') 2, 1:10,000, PI-31403; Pierce, Rockford, IL, USA) for 1 h, and the antibody reactivity was visualized with ECL Western blotting substrate (PI-32106, Pierce) using Kodak Biomax films (#1788207, Sigma Chemicals Co.). Protein bands were quantified using ELLIPSE software version 2.0.7.1 (ViDiTo, SR).

### 2.8. RNA Isolation, Reverse Transcription and Quantitative RT-PCR

Total RNA was isolated using Trizol (Gibco/BRL., Gaithersburg, MD, USA) and purified using RNasy Mini Kit (Qiagen, Hilden, Germany). The RNA concentration was quantified at 260 nm and 1 μg of RNA was transcribed using Superscript II (Invitrogen Co.) reverse transcriptase and oligodT primers (Invitrogen Co.). Quantitative RT-PCRs were performed in duplicates by iCycler iQ Real-Time PCR Detection System (BioRad, Hercules, CA, USA) in 30 μL reaction volume containing: 1× iQ™ SYBR Green Supermix (0.2 mM dNTP, 3 mM MgCl_2_), 0.5 μM forward and reverse primer and 2 μL of cDNA. The reaction conditions were as follows: 95 °C 3 min, 40 cycles (94 °C 30 s, 55 °C 50 s, 72 °C 50 s), 72 °C 7 min followed by melting curve analysis to confirm amplification of the desired single and specific product. The relative expression levels of HER2 (forward primer: 5′-CCT CTG ACG TCC ATC GTC TC-3′ and reverse primer: 5′-CGG ATC TTC TGC TGC CGT CG-3′) and ß-actin (forward primer: 5′-ACC AAC TGG GAC GAC ATG GAG AAA ATC-3′ and reverse primer: 5′-GTA GCC GCG CTC GGT GAG GAT CTT CAT-3′) genes were evaluated using the standard curve method. Standard curves for HER2 and ß-actin were obtained by amplification of serially-diluted mixtures of cDNA samples (four-fold dilutions), with four to five dilution points, each in duplicate. The calculated resulting relative expression of HER2 gene was normalized to relative ß-actin expression (HER2/ß-actin). The results were evaluated as a ratio to untreated control and are presented as mean ± standard deviation of three independent experiments.

### 2.9. Statistical Analysis

Data were processed using scientific graphing and ORIGIN analysis software (OriginLab Co., Northampton, MA, USA) and statistically analyzed using one-way ANOVA followed by Tukey's multiple comparison tests.

## 3. Results and Discussion

Based on the MTT screening data (not shown) we determined the concentrations of 17-DMAG inhibiting 10% (IC_10_) and 50% (IC_50_) of metabolic activity of SKBR-3 cells. These concentrations were further analyzed from SKBR-3 cell proliferation, cell cycle progression, cell signalization and protein changes point of view. In this regard, we have studied the effect of single 17-DMAG as well as its combination with hypericin-PDT. Although, single IC_10_ concentration (5 nM) of 17-DMAG did not significantly influence survival and/or proliferation of SKBR-3 cells during 24 h period (not shown), the effect of this concentration was clearly seen at the 48 h time point.

**Figure 1.**
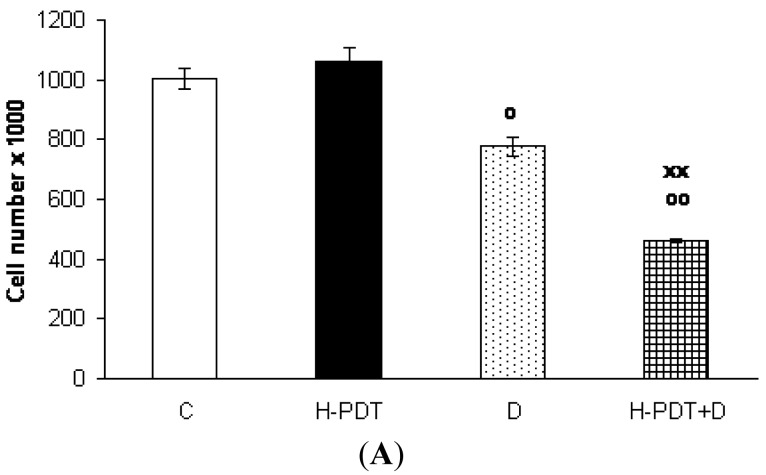

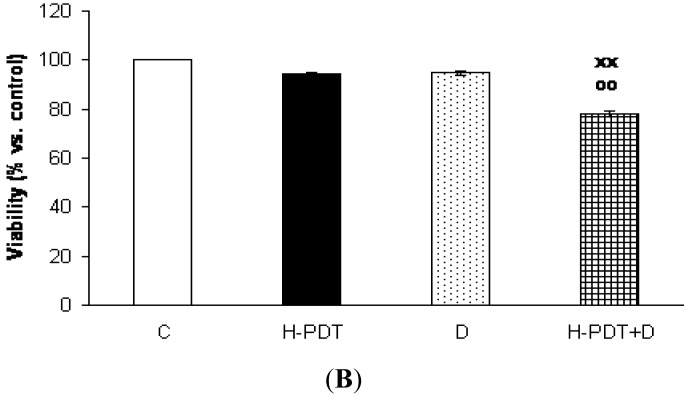
The effect of PDT with hypericin (H-PDT), 17-DMAG (D) and their combination (H-PDT+D) on cell number (**A**) and the viability (**B**) of SKBR-3 cells. The cells were untreated (C) or treated for 24 h with hypericin (21 nM), 17-DMAG (5 nM) or their combination under dark conditions, photoactivated and consequently analyzed 24 h later.

**Figure 2.**
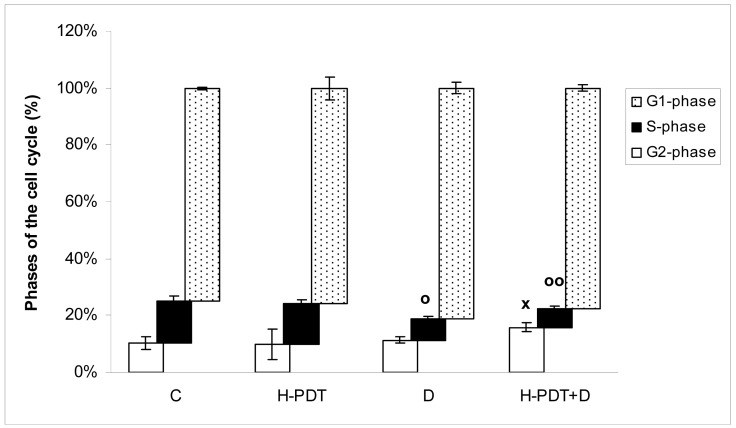
The effect of PDT with hypericin (H-PDT), 17-DMAG (D) and their combination (H-PDT + D) on the cell cycle progression of SKBR-3 cells. The cells were untreated (C) or treated for 24 h with hypericin (21 nM), 17-DMAG (5 nM) or their combination under dark conditions, photoactivated and consequently analyzed 24 h later.

Indeed, 48 h incubation of SKBR-3 cells with 5 nM of 17-DMAG revealed cell number decline (Figure 1A) as well as G1 phase inhibition (Figure 2) of cells.

**Figure 3.**
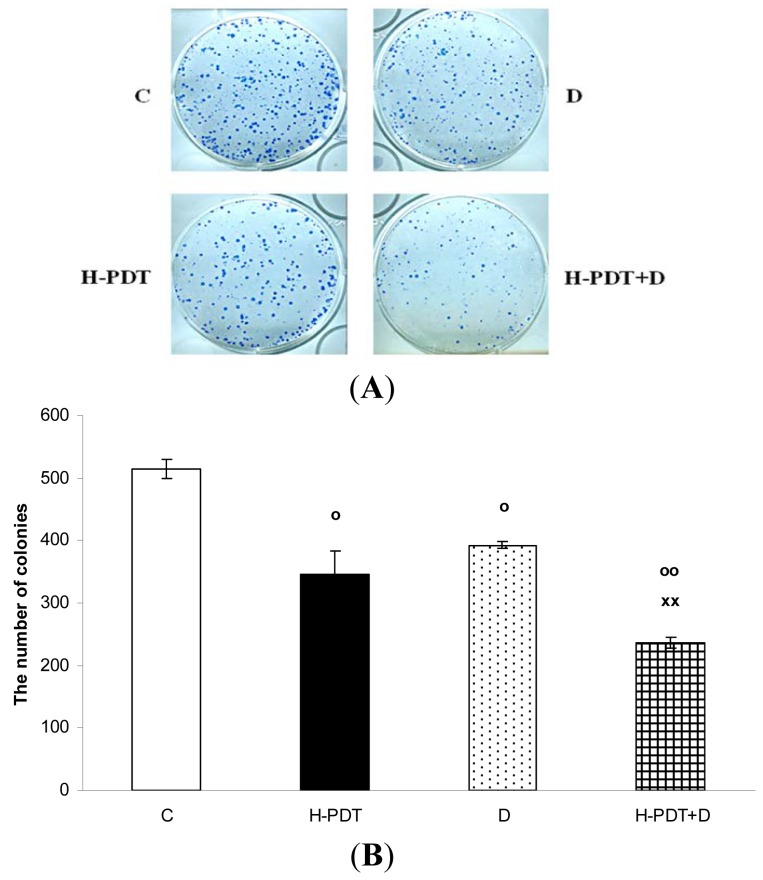
The effect of PDT with hypericin (H-PDT), 17-DMAG (D) and their combination (H-PDT + D) on the clonogenic ability of SKBR-3 cells. The cells were untreated (C) or treated for 24 h with hypericin (21 nM), 17-DMAG (5 nM) or their combination under dark conditions, photoactivated, harvested 24 h later and consequently 500 cells were taken for clonogenic assay. The cells were allowed to grow for 10 days in culture conditions until visible colonies were observed.

Interestingly, markedly reduced clonogenic ability of SKBR-3 cells (Figure 3) together with insignificant cell viability changes (Figure 1B) supported expected anti-proliferating activity of single 17-DMAG.

**Figure 4.**
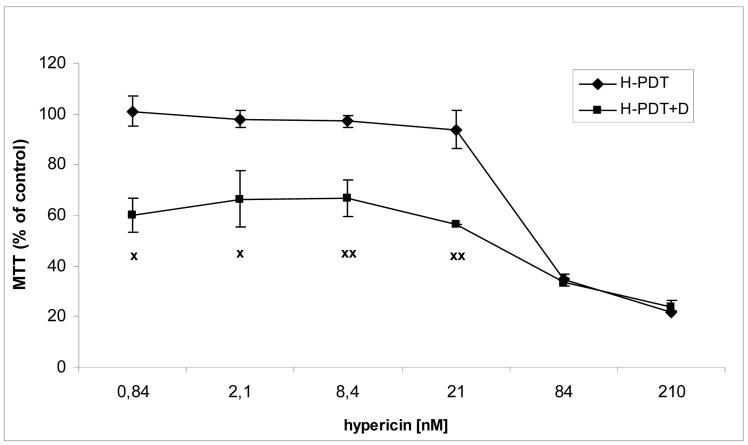
The effect of PDT with hypericin (H-PDT) and its combination with 17-DMAG (H-PDT + D) on the metabolic activity of SKBR-3 cells evaluated by MTT assay. The cells were treated for 24 h with single hypericin (0.84–210 nM) or combined with 17-DMAG (5 nM) under dark conditions, photoactivated and consequently analyzed 48 h later.

**Figure 5.**
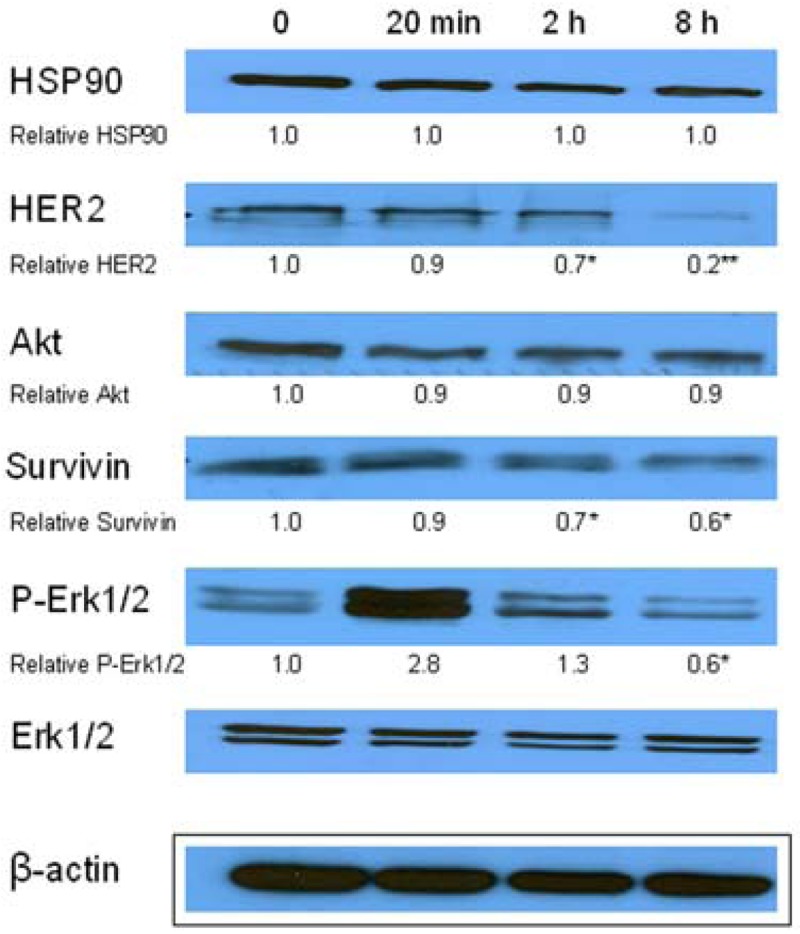
The effect of 17-DMAG treatment on HSP90, HER2, Akt, Survivin and P-Erk1/2 protein levels of SKBR-3 cells in different time after 17-DMAG. Total cell lysates were prepared 0, 20 min, 2 h or 8 h after 17-DMAG (19 nM) treatment. Equal loading was confirmed by detection of beta actin expression. The relative amounts (densitometric levels) of HSP90, HER2, Akt, Survivin and P-Erk1/2 were normalized to beta actin and Erk1/2, respectively.

**Figure 6.**
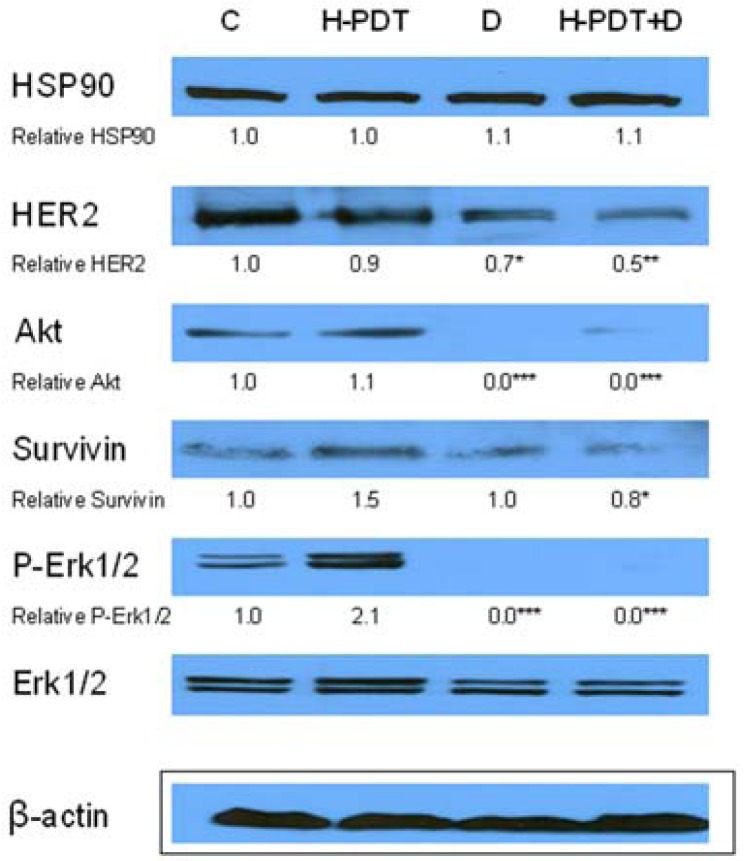
The effect of PDT with hypericin (H-PDT), 17-DMAG (D) and their combination (H-PDT+D) on HSP90, HER2, Akt, Survivin and P-Erk1/2 protein levels of SKBR-3 cells. The cells were untreated (C) or treated for 24 h with hypericin (21 nM), 17-DMAG (5 nM) or their combination under dark conditions, photoactivated and consequently total cell lysates prepared 24 h later. Equal loading was confirmed by detection of beta actin expression. The relative amounts (densitometric levels) of HSP90, HER2, Akt, Survivin and P-Erk1/2 were normalized to beta actin and Erk1/2, respectively.

Similarly, single IC_10_ concentration of hypericin did not change significantly the cell number and the viability of SKBR-3 cells but co-treatment of such a concentration with IC_10_ of 17-DMAG did decrease both cell number as well as the viability of cells *vs.* control and *vs.* single 17-DMAG (Figure 1A, B). Moreover, the combination of both therapies induced pronounced block of cells in G2 phase of the cell cycle *vs.* control (Figure 2). The effectiveness of hypericin-PDT combined with 17-DMAG was also shown in the clonogenic assay and MTT result, where co-treatment markedly reduced SKBR-3 cell proliferation potential and induced more pronounced cytotoxic effect, respectively (Figures 3 and 4). In addition, IC_50_ concentration (19 nM) of 17-DMAG diminished survivin and HER2 protein levels already 2 h after 17-DMAG application. Interestingly, 19 nM concentration of 17-DMAG induced initial increase in phosphorylation of Erk1/2 observed from 20 min to 2 h followed by its drop at 8 h after 17-DMAG treatment (Figure 5). Indeed, there was a positive correlation between reduced clonogenic ability of SKBR-3 cells and HER2, Akt and P-Erk1/2 protein decline observed 48 h after 17-DMAG administration (Figure 6).

Hypothesized HER2 protein inhibition of co-treatment *vs.* individual therapies was demonstrated by Western blot results where HER2 degradation was more significant after combined treatment than after individual ones (Figure 6). Except for HER2 protein degradation we could observe a drop of survivin protein which resulted more pronounced from the combination of 17-DMAG and hypericin-PDT (Figure 6). In regard to hypericin-PDT effects we found increased protein levels of P-Erk1/2 and survivin changes (Figure 6) which are discussed below.

**Figure 7.**
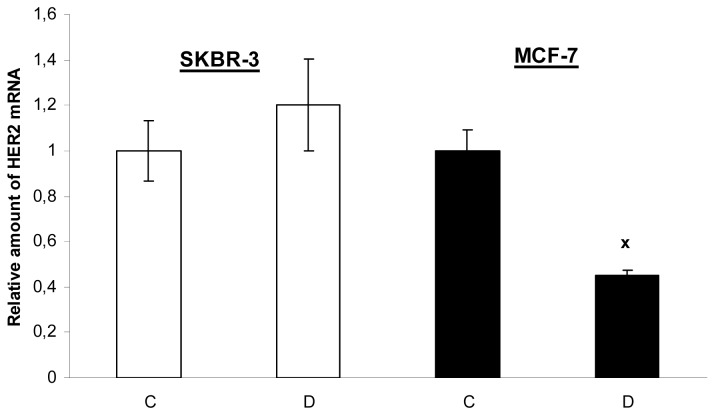
Relative amounts of HER2 mRNA in SKBR-3 and MCF-7 cells after 17-DMAG treatment. SKBR-3 and MCF-7 cells were untreated (C) or treated with 17-DMAG (19 nM) (D) for 24 h and taken for RNA isolation. Expression of HER2 gene was normalized to β-actin expression (HER2/β-actin) and is shown as a ratio to C.

The novel finding of our study is that 17-DMAG decreased the mRNA level of HER2 but only in MCF-7 cells with low expression of HER2, but not in HER2 overexpressed SKBR-3 cells (Figure 7). The mechanism of 17-DMAG induced HER2 gene downregulation in MCF-7 cells will be analyzed more detailed in our next study.

Cells respond to environmental stress by increasing synthesis of several molecular chaperons known as heat shock proteins (HSP). These proteins are categorized according to their molecular weight into five classes: small HSP, HSP60, HSP70, HSP90 and HSP100.

One of the most abundant molecular chaperones is HSP90, which is a highly conserved protein whose association is required for the stability and function of multiple-mutated, chimeric- and over-expressed-signaling proteins that promote the growth and/or survival of cancer cells [25]. Many of these proteins have been identified, and the list now includes proteins such as Raf-1 kinase [26], mutated p53 [27], Akt kinase [28], Bcr-Abl kinase [29], hypoxia-inducible factor 1 alpha [30] and others. We have shown that cisplatin-resistant CP70 and C200 cells, prepared by prolonged cisplatin treatment of parental A2780 cells, have significantly higher expression of HSPCA (HSP90 alpha) and TRA1 (GRP94). Over-expressed GRP94 and HSP90 alpha might protect CP70 and C200 cells from cisplatin toxicity and render them more resistant [31]. Geldanamycin a natural protein isolated from *Streptomyces hygroscopicus*, bound specifically to HSP90 and inhibited the association of the chaperone with oncogenic protein kinases via its proteasomal degradation [32]. Our current study confirmed cytostatic effect of geldanamycin derivate 17-DMAG on HER2 over-expressed breast adenocarcinoma cell line SKBR-3.

In this regard, Wang *et al.* [12] demonstrated that geldanamycin reduced the tumorigenicity of SKBR-3 cells, partly by destabilizing the HER2 oncoprotein, and by disruption of the functional relationship between HER2 and the Wnt/β-catenin signaling pathways. HSP90 associated proteins were also found to be diminished in our presented study, when single 17-DMAG induced HER2, Akt and survivin degradation and down-regulation of Erk1/2 signalization without disruption of Erk1/2 proteins. The effect of decreased Erk1/2 phosphorylation resulted in our study probably from proteasomal or lysosomal degradation of upstream protein Raf-1, which is one of the HSP90 client proteins. Indeed, 17-DMAG induced proteasomal degradation of Raf-1 and Akt followed by the inhibition of FGF-2 or VEGF stimulated HUVEC cell proliferation [33]. Furthermore, Palacios *et al.* [34] demonstrated that treatment of human breast cancer cells with 17-DMAG caused degradation of RIP1 kinase and promoted the activation of a mitochondria-operated, caspase-dependent mechanism of apoptosis in these cells.

The combination of geldanamycin derivate 17-AAG and monoclonal antibody against HER2 protein-trastuzumab treatments led to enhanced lysosomal degradation of HER2 protein and induced synergistic growth arrest and cell death of HER2 over-expressing breast cancer cell lines [13]. Moreover, quantitative microPET imaging of Niu *et al.* [14] showed that ^60^Cu-DOTA-trastuzumab had prominent tumor accumulation in untreated SKOV-3 tumors, which was significantly reduced in 17-DMAG-treated ones.

In our previous study, cisplatin-resistant derivatives of A2780 cells pretreated with geldanamycin showed two times higher sensitivity to cisplatin treatment [35]. Currently, 17-DMAG revealed potentiation effect on SKBR-3 cells response to hypericin-PDT. The combination of 17-DMAG and hypericin-PDT, inspite of unsignificant effect of single IC_10_ concentration (21 nM) of hypericin, resulted in markedly destabilization of HER2 protein in comparison to single 17-DMAG treatment. This result correlated well with cell survival and/or proliferation regress of SKBR-3 cells. On the other hand, any significant effect of combination *vs.* 17-DMAG was seen in depletion of Akt and P-Erk1/2 likely because of very effective HSP90 inhibition of IC_10_ concentration of 17-DMAG alone.

Recently, Ferenc *et al.* [36] demonstrated Akt and Erk1/2 phosphorylation effect after low doses of hypericin-PDT, while higher concentrations had an opposite effect [22]. In addition to the modulation of Akt and Erk1/2 signaling pathways, PDT increased the expression of the anti-apoptotic and pro-angiogenic proteins survivin, HIF-1α, MMP-2 and VEGF in tumor tissue and this expression decreased significantly when 17-AAG was included in the treatment regimen [37]. In accordance to Ferenc *et al.* [36] and Ferrario *et al.* [37] we found increased P-Erk1/2 and survivin levels, respectively, after hypericin-PDT in SKBR-3 cells. In combination, 17-DMAG protected SKBR-3 cells against both P-Erk1/2 as well as survivin up-regulations induced by hypericin-PDT.

Nowadays, phase I study of the 17-DMAG administered twice weekly to patients with acute myeloid leukemia [38] and patients with advanced cancer [39] was well tolerated, showing linear pharmacokinetics, target inhibition and signs of clinical activity.

## 4. Conclusions

Our results show that by targeting HSP90 client proteins we can increase the efficiency of antineoplastic effect of photodynamic therapy *in vitro*. Based on published as well as our current results we hypothesize that the combination of hypericin-PDT and 17-DMAG could have a great impact in clinical applications at least in the treatment of chest wall recurrence of HER2 positive breast carcinoma.

## Figures and Tables

Data are presented as means ± SD of three independent experiments. The statistical significance is designated as follows: H-PDT+D or D *vs.* C ^o^ *P* < 0.05 or ^oo^ *P* < 0.01; H-PDT+D *vs.* D ^xx^ *P* < 0.01.

Data are presented as means ± SD of three independent experiments. The statistical significance is designated as follows: H-PDT+D or D *vs.* C ^o^ *P* < 0.05 or ^oo^ *P* < 0.01 (S-phase); H-PDT+D *vs.* C ^x^ *P* < 0.05 (G2-phase).

Data are presented as representative picture of colonies (**A**) and as means of colonies ± SD of three independent experiments (**B**). The statistical significance is designated as follows: H-PDT, D or H-PDT+D *vs.* C ^o^ *P* < 0.05 or ^oo^ *P* < 0.01; H-PDT+D *vs.* D ^xx^ *P* < 0.01.

The results were evaluated as percentage of untreated control and are presented as means ± SD of three independent experiments. The statistical significance is designated as follows: H-PDT+D *vs.* H-PDT ^x^ *P* < 0.05 or ^xx^ *P* < 0.01.

Data are presented as representative picture of three western blots and as means of densities taken from three independent experiments. The statistical significance is designated as follows: 2 h or 8 h *vs.* 0 ^*^ *P* < 0.05 or ^**^ *P* < 0.01.

Data are presented as representative picture of three Western blots and as means of densities taken from three independent experiments. The statistical significance is designated as follows: H-PDT+D or D *vs.* C ^*^ *P* < 0.05, ^**^ *P* < 0.01 or ^***^ *P* < 0.001.

Data are presented as means ± SD of three independent experiments. The statistical significance is designated as follows: D *vs.* C ^x^ *P* < 0.05.

## Abbreviations

17-AAG: 17-allylamino-17-demethoxygeldanamycin
17-DMAG: 17-dimethylaminoethylamino-17-demethoxygeldanamycin
A2780: human ovarian adenocarcinoma cell line
Akt: protein kinase
Bcr-Abl: oncogene fusion protein (tyrosine kinase)
ECL: enhanced chemiluminescence
Erk1/2: extracellular signal-regulated kinase 1/2
FCS: fetal calf serum
FGF-2: fibroblast growth factor 2
HER2: human epidermal growth factor receptor 2
HIF-1α: hypoxia-inducible factor 1 alpha
HSP: heat shock protein (molecular chaperones)
HUVEC: human umbilical vein endothelial cells
IC_10_: the concentration inhibiting 10% of cells metabolic activity
IC_50_: the concentration inhibiting 50% of cells metabolic activity
IGF-IR: insulin-like growth factor receptor
IgG: immunoglobulin G
MAP: mitogen activated protein kinase
MCF-7: human breast adenocarcinoma cell line
MMP-2: matrix metalloproteinase 2
mTOR: mammalian target of rapamycin
MTT: 3-(4.5-dimethylthiazol-2-yl)-2.5-diphenyl tetrazolium bromide
MUC4: mucin 4
OD: optical density
PBS: phosphate-buffered saline
PDT: photodynamic therapy
P-Erk1/2: phosphorylated form of extracellular signal-regulated kinase 1/2
PI3K: phosphoinositide 3-kinase
PTEN: phosphatase and tensin homolog
Raf-1: protein kinase
RIP1: receptor-interacting serine-threonine kinase
RT-PCR: reverse transcriptase PCR
SD: standard deviation
SDS: sodium dodecyl sulfate
SKBR-3: human breast adenocarcinoma cell line
TKI: tyrosine kinase inhibitor
TRA1: tumor rejective antigen 1
VEGF: vascular endothelial growth factor

## References

[1] Nahta R., Yu D., Hung M.C., Hortobagyi G.N., Esteva F.J. (2006). Mechanisms of disease: Understanding resistance to HER2-targeted therapy in human breast cancer. Nat. Clin. Pract. Oncol..

[2] Haffty B.G., Hauser A., Choi D.H., Parisot N., Rimm D., King B., Carter D. (2004). Molecular markers for prognosis after isolated postmastectomy chest wall recurrence. Cancer.

[3] Pegram M.D., Pienkowski T., Northfelt D.W., Eiermann W., Patel R., Fumoleau P., Quan E., Crown J., Toppmeyer D., Smylie M. (2004). Results of two open-label, multicenter phase II studies of docetaxel, platinum salts, and trastuzumab in HER2-positive advanced breast cancer. J. Natl. Cancer Inst..

[4] Perez E.A., Suman V.J., Rowland K.M., Ingle J.N., Salim M., Loprinzi C.L., Flynn P.J., Mailliard J.A., Kardinal C.G., Krook J.E. (2005). Two concurrent phase II trials of paclitaxel/carboplatin/trastuzumab (weekly or every-3-week schedule) as first-line therapy in women with HER2-overexpressing metastatic breast cancer: NCCTG study 983252. Clin. Breast Cancer.

[5] Robert N., Leyland-Jones B., Asmar L., Belt R., Ilegbodu D., Loesch D., Raju R., Valentine E., Sayre R., Cobleigh M. (2006). Randomized phase III study of trastuzumab, paclitaxel, and carboplatin compared with trastuzumab and paclitaxel in women with HER-2-overexpressing metastatic breast cancer. J. Clin. Oncol..

[6] Nagata Y., Lan K.H., Zhou X., Tan M., Esteva F.J., Sahin A.A., Klos K.S., Li P., Monia B.P., Nguyen N.T. (2004). PTEN activation contributes to tumor inhibition by trastuzumab, and loss of PTEN predicts trastuzumab resistance in patients. Cancer Cell.

[7] Lu Y., Zi X., Zhao Y., Mascarenhas D., Pollak M. (2001). Insulin-like growth factor-I receptor signaling and resistance to trastuzumab (herceptin). J. Natl. Cancer Inst..

[8] Nagy P., Friedlander E., Tanner M., Kapanen A.I., Carraway K.L., Isola J., Jovin T.M. (2005). Decreased accessibility and lack of activation of ErbB2 in JIMT-1, a herceptin-resistant, MUC4-expressing breast cancer cell line. Cancer Res..

[9] du Manoir J.M., Francia G., Man S., Mossoba M., Medin J.A., Viloria-Petit A., Hicklin D.J., Emmenegger U., Kerbel R.S. (2006). Strategies for delaying or treating *in vivo* acquired resistance to trastuzumab in human breast cancer xenografts. Clin. Cancer Res..

[10] Morrow P.K., Zambrana F., Esteva F.J. (2009). Recent advances in systemic therapy: Advances in systemic therapy for HER2-positive metastatic breast cancer. Breast Cancer Res..

[11] Castilleja A., Ward N.E., O'Brian C.A., Swearingen B., Swan E., Gillogly M.A., Murray J.L., Kudelka A.P., Gershenson D.M., Ioannides C.G. (2001). Accelerated HER-2 degradation enhances ovarian tumor recognition by CTL. Implications for tumor immunogenicity. Mol. Cell. Biochem..

[12] Wang K., Ma Q., Ren Y., He J., Zhang Y., Zhang Y., Chen W. (2007). Geldanamycin destabilizes HER2 tyrosine kinase and suppresses Wnt/beta-catenin signaling in HER2 overexpressing human breast cancer cells. Oncol. Rep..

[13] Raja S.M., Clubb R.J., Bhattacharyya M., Dimri M., Cheng H., Pan W., Ortega-Cava C., Lakku-Reddi A., Naramura M., Band V. (2008). A combination of trastuzumab and 17-AAG induces enhanced ubiquitinylation and lysosomal pathway-dependent ErbB2 degradation and cytotoxicity in ErbB2-overexpressing breast cancer cells. Cancer Biol. Ther..

[14] Niu G., Li Z., Cao Q., Chen X. (2009). Monitoring therapeutic response of human ovarian cancer to 17-DMAG by noninvasive PET imaging with (64)CU-DOTA-trastuzumab. Eur. J. Nucl. Med. Mol. Imaging.

[15] Munster P.N., Marchion D.C., Basso A.D., Rosen N. (2002). Degradation of HER2 by ansamycins induces growth arrest and apoptosis in cells with HER2 overexpression via a HER3, phosphatidylinositol 3′-kinase-Akt-dependent pathway. Cancer Res..

[16] Modi S., Stopeck A.T., Gordon M.S., Mendelson D., Solit D.B., Bagatell R., Ma W., Wheler J., Rosen N., Norton L. (2007). Combination of trastuzumab and tanespimycin (17-AAG, KOS-953) is safe and active in trastuzumab-refractory HER-2 overexpressing breast cancer: A phase I dose-escalation study. J. Clin. Oncol..

[17] Modi S., Stopeck A., Linden H., Solit D., Chandarlapaty S., Rosen N., D'Andrea G., Dickler M., Moynahan M.E., Sugarman S. (2011). Hsp90 inhibition is effective in breast cancer: A phase II trial of tanespimycin (17-AAG) plus trastuzumab in patients with HER2-positive metastatic breast cancer progressing on trastuzumab. Clin. Cancer Res..

[18] Wilson B.C. (2002). Photodynamic therapy for cancer: Principles. Can. J. Gastroenterol..

[19] Duran N., Song P.S. (1986). Hypericin and its photodynamic action. Photochem. Photobiol..

[20] Allison R., Mang T., Hewson G., Snider W., Dougherty D. (2001). Photodynamic therapy for chest wall progression from breast carcinoma is an underutilized treatment modality. Cancer.

[21] Cuenca R.E., Allison R.R., Sibata C., Downie G.H. (2004). Breast cancer with chest wall progression: Treatment with photodynamic therapy. Ann. Surg. Oncol..

[22] Solar P., Ferenc P., Koval J., Mikes J., Solarova Z., Hrckova G., Fulton B.L., Fedorocko P. (2011). Photoactivated hypericin induces downregulation of HER2 gene expression. Radiat. Res..

[23] Koval J., Mikes J., Jendzelovsky R., Kello M., Solar P., Fedorocko P. (2010). Degradation of HER2 receptor through hypericin-mediated photodynamic therapy. Photochem. Photobiol..

[24] Niyazi M., Niyazi I., Belka C. (2007). Counting colonies of clonogenic assays by using densitometric software. Radiat. Oncol..

[25] Neckers L. (2002). Hsp90 inhibitors as novel cancer chemotherapeutic agents. Trends Mol. Med..

[26] Schulte T.W., Blagosklonny M.V., Romanova L., Mushinski J.F., Monia B.P., Johnston J.F., Nguyen P., Trepel J., Neckers L.M. (1996). Destabilization of Raf-1 by geldanamycin leads to disruption of the Raf-1-MEK-mitogen-activated protein kinase signalling pathway. Mol. Cell. Biol..

[27] Blagosklonny M.V., Toretsky J., Bohen S., Neckers L. (1996). Mutant conformation of p53 translated *in vitro* or *in vivo* requires functional HSP90. Proc. Natl. Acad. Sci. USA.

[28] Sato S., Fujita N., Tsuruo T. (2000). Modulation of Akt kinase activity by binding to HSP90. Proc. Natl. Acad. Sci. USA.

[29] An W.G., Schulte T.W., Neckers L.M. (2000). The heat shock protein 90 antagonist geldanamycin alters chaperone association with p210^bcr-abl^ and v-src proteins before their degradation by the proteasome. Cell Growth Differ..

[30] Minet E., Mottet D., Michel G., Roland I., Raes M., Remacle J., Michiels C. (1999). Hypoxia-induced activation of HIF-1: Role of HIF-1alpha-Hsp90 interaction. FEBS Lett..

[31] Solar P., Sytkowski A.J. (2011). Differentially expressed genes associated with cisplatin resistance in human ovarian adenocarcinoma cell line A2780. Cancer Lett..

[32] Pratt W.B., Toft D.O. (2003). Regulation of signaling protein function and trafficking by the hsp90/hsp70-based chaperone machinery. Exp. Biol. Med. (Maywood).

[33] Kaur G., Belotti D., Burger A.M., Fisher-Nielson K., Borsotti P., Riccardi E., Thillainathan J., Hollingshead M., Sausville E.A., Giavazzi R. (2004). Antiangiogenic properties of 17-(dimethylaminoethylamino)-17-demethoxygeldanamycin: An orally bioavailable heat shock protein 90 modulator. Clin. Cancer Res..

[34] Palacios C., Lopez-Perez A.I., Lopez-Rivas A. (2010). Down-regulation of RIP expression by 17-dimethylaminoethylamino-17-demethoxygeldanamycin promotes TRAIL-induced apoptosis in breast tumor cells. Cancer Lett..

[35] Solar P., Horvath V., Kleban J., Koval J., Solarova Z., Kozubik A., Fedorocko P. (2007). Hsp90 inhibitor geldanamycin increases the sensitivity of resistant ovarian adenocarcinoma cell line A2780cis to cisplatin. Neoplasma.

[36] Ferenc P., Solar P., Kleban J., Mikes J., Fedorocko P. (2010). Down-regulation of Bcl-2 and Akt induced by combination of photoactivated hypericin and genistein in human breast cancer cells. J. Photochem. Photobiol. B.

[37] Ferrario A., Gomer C.J. (2010). Targeting the 90 kDa heat shock protein improves photodynamic therapy. Cancer Lett..

[38] Lancet J.E., Gojo I., Burton M., Quinn M., Tighe S.M., Kersey K., Zhong Z., Albitar M.X., Bhalla K., Hannah A.L. (2010). Phase I study of the heat shock protein 90 inhibitor alvespimycin (KOS-1022, 17-DMAG) administered intravenously twice weekly to patients with acute myeloid leukemia. Leukemia.

[39] Kummar S., Gutierrez M.E., Gardner E.R., Chen X., Figg W.D., Zajac-Kaye M., Chen M., Steinberg S.M., Muir C.A., Yancey M.A. (2010). Phase I trial of 17-dimethylaminoethylamino-17-demethoxygeldanamycin (17-DMAG), a heat shock protein inhibitor, administered twice weekly in patients with advanced malignancies. Eur. J. Cancer.

